# Involvement of Myofascial Spiral Chains of the Lower Limb in Semi-unipodal Balance: A Pilot Study

**DOI:** 10.7759/cureus.42468

**Published:** 2023-07-26

**Authors:** Corrado Borghi, Saverio Colonna, Francesco Lombardi

**Affiliations:** 1 Children Rehabilitation Unit - UDGEE, Azienda USL-IRCCS di Reggio Emilia, Reggio Emilia, ITA; 2 Ostheopathic Spine Center Education, Spine Center, Bologna, ITA; 3 Neurorehabilitation Unit, Azienda USL-IRCCS di Reggio Emilia, Reggio Emilia, ITA

**Keywords:** motor control, electromyography, monopodal stance, single-leg balance, spiral chains, myofascial chains

## Abstract

Introduction

Single-leg stance has been extensively studied for functional evaluation, therapeutic exercise, sports training, and fall prevention. However, the motor strategies of the supporting limb have been investigated only at the ankle level. It is not known, at the hip, how the muscular system reacts to medial and lateral imbalances. We hypothesize, based on a myofascial chain approach, that the balance is managed by the front and back spiral chains. The aim of this work was to perform a preliminary experimental analysis to verify the spiral chain hypothesis, testing a method to investigate the motor strategies underlying equilibrium.

Methods

Five healthy subjects (i.e. without neurological or orthopedic pathologies affecting the upright position) underwent perturbations of their monopodal balance while a surface electromyographic analysis of gluteus maximus, gluteus medius, adductor longus (ADD), tibialis anterior (TA), and peroneus longus (PL) was executed. The percentage of electrical activation with respect to maximal contraction was calculated for each muscle investigated. The coordination in activation between the hip and ankle muscles was analyzed by the Pearson correlation coefficient.

Results

Of the studied muscles, TA (43% of maximal contraction) and gluteus medius (28%) had the average highest reaction to lateral imbalance and the highest correlation coefficient (0.89, p-value<0.01); PL (35%) and ADD (16%) were the most relevant in counteracting the medial imbalance (correlation coefficient=0.83, p-value<0.01).

Conclusion

The study was performed on a few subjects, and the muscles of the lower limb were only partially investigated. However, the consistency of the results with former experimental studies provided preliminary evidence of the adequacy of the method adopted. The correlation of hip and ankle muscle activations was in line with the spiral chain hypothesis.

## Introduction

A body, including that of humans, is in balance when the vertical projection of the center of gravity falls into the base of support. Human balance strategies have been widely researched but have not yet been definitively clarified.

The best-known strategies mainly concern bipodalic equilibrium. In this condition, the base of support is generally narrower in the antero-posterior direction. Along this direction, the vertical projection of the center of gravity can fall more easily out of the base of support. Therefore, the plane of greatest interest is the sagittal one. In this plane, several authors identified and described the so-called “ankle-strategy,” “hip-strategy,” and “stepping-strategy” [1-5].

Monopodalic balance has been less addressed. It is much more critical as the base of support is limited to a single footprint. If the foot is fully leaning on the ground, the footprint is narrower in the medio-lateral direction. In this case, the main imbalances can be observed in the frontal plane. MacKinnon et al. studied the balance control on a single foot during walking [6]. They defined an inverted pendulum model and described the equilibrium as a balance of moments of force: moments at the ankle and hip equal the inertial moment of force of the whole system. Otten further reduced the base of support to a narrow ridge [7]. He identified the contribution of each main body segment to achieve the monopodalic balance. Through simulation and measurements of the kinematics and dynamics, he pointed out the fundamental role of the hips. In particular, he determined the relationship between hip moments and correction of imbalance in a specific direction: hip adduction moment of the supporting leg compensates for a medial imbalance, bringing the center of mass laterally; hip abduction moment works conversely.

The actual muscle contribution and timing of activation remain unclear. A large number of studies utilized the electromyographic (EMG) analysis to describe the latency and intensity of activation in many different balance tasks [8-15]. Still, to our knowledge, only Morey-Klapsing et al. distinguished the muscle response to medial vs lateral imbalances in a one-legged stance [16]. Their analysis focused on ankle stabilization on a tilting platform and therefore was limited to ankle muscles. The tip of the free leg was allowed to touch the ground to help maintain balance and reduce EMG activity prior to tilt. They acknowledged that the peroneus longus (PL) responds to a medial tilt, and the tibialis anterior (TA) to a lateral tilt. No information is available about the hip joint muscles.

Colonna advanced the hypothesis that the stabilization of the supporting leg is determined by a “cross system” [17]. The basic idea is that a structure with crossing elements is more stable (i.e., in high-voltage pylons or in building cranes). These crossing elements consist of “myofascial chains” [17, 18]. The myofascial chains concept suggests that the mechanical force can be transferred through the connective tissue not only within a limb between synergistic and antagonistic muscles but also between muscles arranged in series [19]. While force transfer is still debated, there are many proofs of the anatomical continuity of fascial chains [20, 21].

The approach proposed by Colonna contemplates eight systems of chains [17]: lateral static, posterior static-dynamic, anterior static-dynamic, flexion, extension, lateral, anterior spiral, and posterior spiral. On the frontal plane, the chains with a configuration able to influence the medio-lateral balance are the anterior spiral, the posterior spiral, and the lateral. The main muscles of the lower limbs that participate in the aforementioned chains are the following: for the anterior spiral chain are oblique and transverse abductor, peronei (short, long, and anterior), external gastrocnemius, the short head of the biceps, adductors (small, medium, and large), pectineus, vastus medialis, gracilis, and tensor fascia latae; for the posterior spiral chain are adductor hallucis, flexor hallucis longus, tibialis posterior, popliteus, vastus lateralis, extensor hallucis longus, TA, the long head of the biceps, piriformis, tensor fascia lata, and glutei (small, medium, and large); and for the lateral static chain are plantar aponeurosis, peronei, TA, tensor fascia lata, glutei, and piriformis.

According to Colonna’s hypothesis, the muscle of the anterior spiral chain of the supporting leg (i.e., eversors of the ankle and adductors of the hip) should activate simultaneously as a response to a medial imbalance [17]. The muscle of the posterior spiral chain (i.e., inversors of the ankle and abductors of the hip) should react to a lateral imbalance.

The aim of this pilot study is to identify the activation of hip and ankle muscles as a response to medial and lateral imbalance during semi-unipodal stance. The objective, more generally, is therefore to identify the synergies of the muscular systems, still unclear on the frontal plane, used to obtain balance at the hip and ankle level.

## Materials and methods

Five subjects voluntarily participated in this pilot study. The sample was not meant to be representative of the population but useful for a preliminary test of the hypothesis. The inclusion criteria were as follows: healthy adults aged between 20 and 60 years, asymptomatic in maintaining an upright posture, and with physiological maintenance of balance. The exclusion criteria were as follows: neurological or orthopedic pathologies capable of compromising upright posture and balance (e.g., knee or hip arthrosis, acute ankle sprains, stroke). The characteristics of the subjects (mean age 34.4, SD 14.5; 80% males, 20% females) are shown in Table 1. They all gave informed consent, and the experimental protocol was approved by the local ethic committee (957/2020/SPER/AUSLRE).

**Table 1:** Characteristics of the five participants.

Subject ID	Age	Gender	Weight (kg)	Height (cm)
S1	23	M	70	173
S2	30	M	74	181
S3	25	M	77	174
S4	35	F	53	158
S5	59	M	73	168

Participants were asked to stand on their bare right foot. Left forefoot was allowed to touch the ground, with the verbal indication to load it the least possible, to reduce EMG activity prior to external perturbations. The maximum accepted load on the left foot was 30% of the body weight. The hands had to be placed on the hips. The left thigh had to be kept vertical. Finally, the subject was required to find, within the constraints described above, a posture that was as comfortable as possible, with minimal muscle contraction. The distance between the feet was therefore freely chosen by each subject.

**Figure 1:**
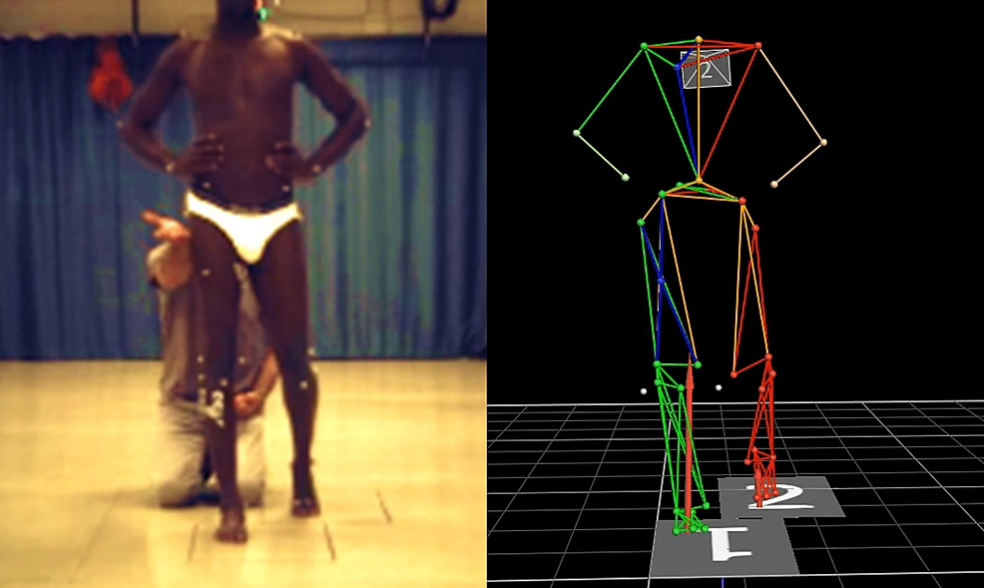
Execution of the push to generate a medial imbalance, with the correspondent 3D representation as acquired by the optoelectronic system.

An operator, the same for all participants, positioned himself behind the subject and, without being seen, gave lateral pushes with the medial margin of the hand at the proximal third of the thigh (Figure 1). At least 10 pushes per side were performed in sequence and with random timing. The pushes were given quickly but without causing pain (the duration of the push was about 0.3-0.4 seconds).

Muscle activation was recorded by a Cometa® surface EMG system, at a sampling rate of 1000 Hz. Bipolar analog surface electrodes with an interelectrode distance of 2 cm were used for EMG collection. EMG signals of TA, PL, gluteus maximum (GMAX), gluteus medium (GMED), and adductor longus (ADD) were acquired. Muscles were chosen for their mechanical relevance in hip ab-adduction (abduction: GMAX, GMED; adduction: ADD) and ankle in-eversion (inversion: TA; eversion: PL) and their representation of posterior (GMAX, GMED, TA) and anterior (ADD, PL) spiral chains. No muscles that did not belong to the spiral chain were analyzed. Deep muscles were excluded for the impossibility to measure their activation by means of surface electromyography.

Maximal isometric contractions for each muscle were measured. The operator provided a manual resistance while requesting a maximal effort for dorsiflexion and pronation of the foot (while sitting) and then for adduction, abduction, and extension of the hip (while standing).

The load on the supporting foot was measured by a force platform (AMTI®, sampling rate of 1000 Hz). The motion of the subjects and the operator was acquired by a Vicon® optoelectronic system (Oxford Metrics Group, UK) equipped with seven cameras (sampling rate of 100 Hz). The marker set applied to the subjects followed the Total3DGait protocol [22]. Two markers were applied to the hand of the operator. The beginning instant of the push was identified as the first instant in which a medio-lateral displacement of the hip was detectable. All the collected signals were automatically synchronized by the acquisition system.

EMG activity was considered in temporal windows of 1500 ms: 500 ms prior to external perturbation and 1000 ms after its beginning. In the first 500 ms, the “resting” activity was observed. The “response” activity, from 500 to 1500 ms, was identified as a modification of the resting activity.

EMG signal underwent the following elaboration procedure: high pass filtering (cutoff frequency 60 Hz, performed with a specific Vicon® software) to reduce movement artifacts; application of root mean square with a 200 ms span to define the signal intensity; and normalization to maximal contraction. For each muscle of each subject, the median among the elaborated signals was computed.

The peaks of the electrical activity of each muscle in response to the perturbations were taken into consideration. The instant at 150 ms after the start of the push was considered as the time reference for the response peaks. The peak value was measured with respect to the resting value before the push.

Finally, Pearson correlation coefficients between ankle and hip muscles were computed to verify the possible synergies. The p-value was set at 0.01 for each correlation coefficient. Microsoft Excel® software was used for the calculations. As regards the statistics of the sample, it should be emphasized that the size of the sample itself was not chosen to obtain statistically or clinically relevant results. For this reason, given the preliminary nature of the study, a proper statistical analysis was not defined. However, to make the results clearer, the mean, standard deviation, and p-values obtained with the t-test have been indicated for the electrical activation peaks.

## Results

**Figure 2:**
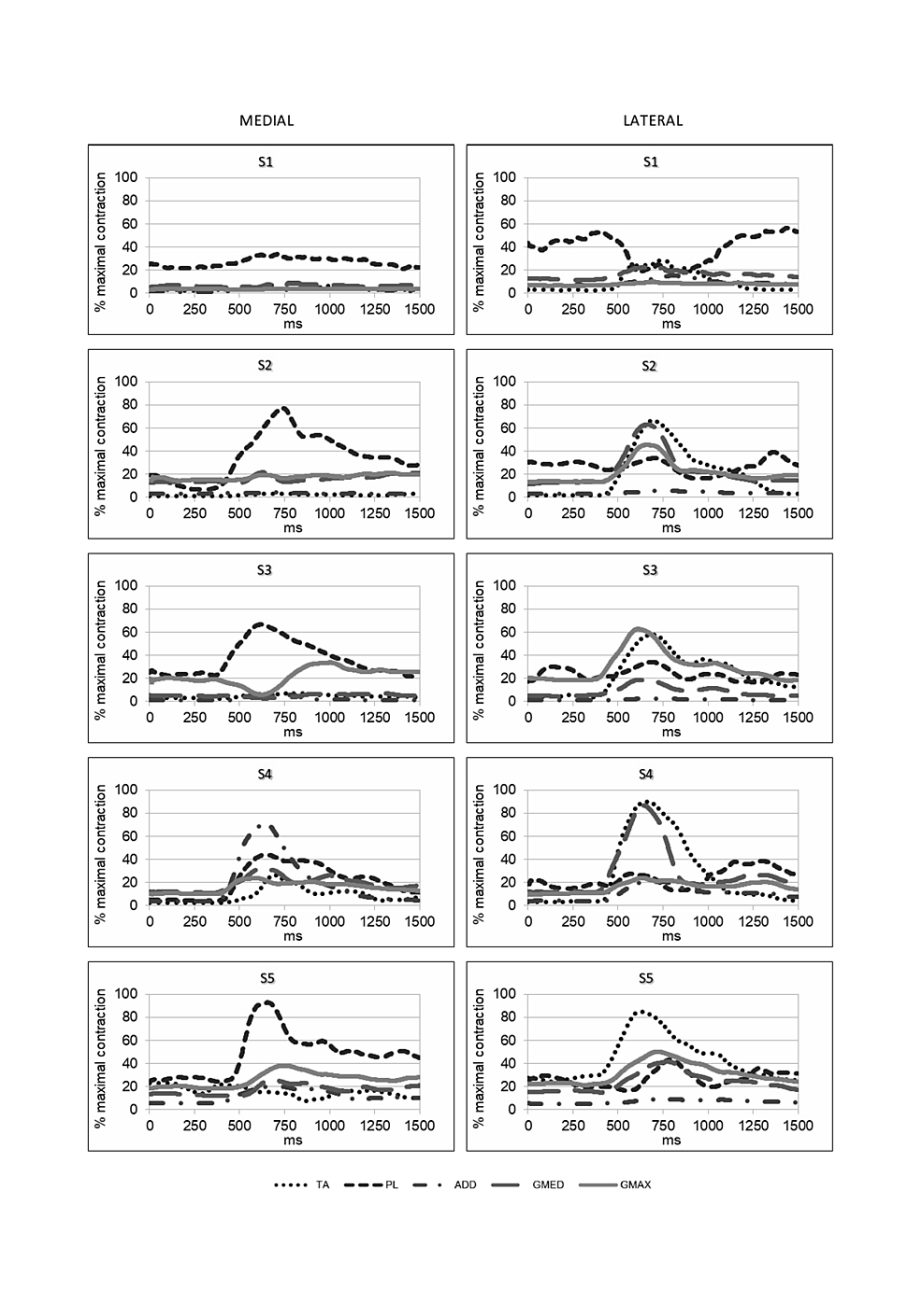
Electrical response to medial and lateral imbalance. The measured muscles (tibialis anterior – TA, peroneus longus – PL, gluteus medius – GMED, gluteus maximus – GMAX, adductor longus – ADD) are presented for each subject.

EMG activation is shown in Figure 2.

**Figure 3:**
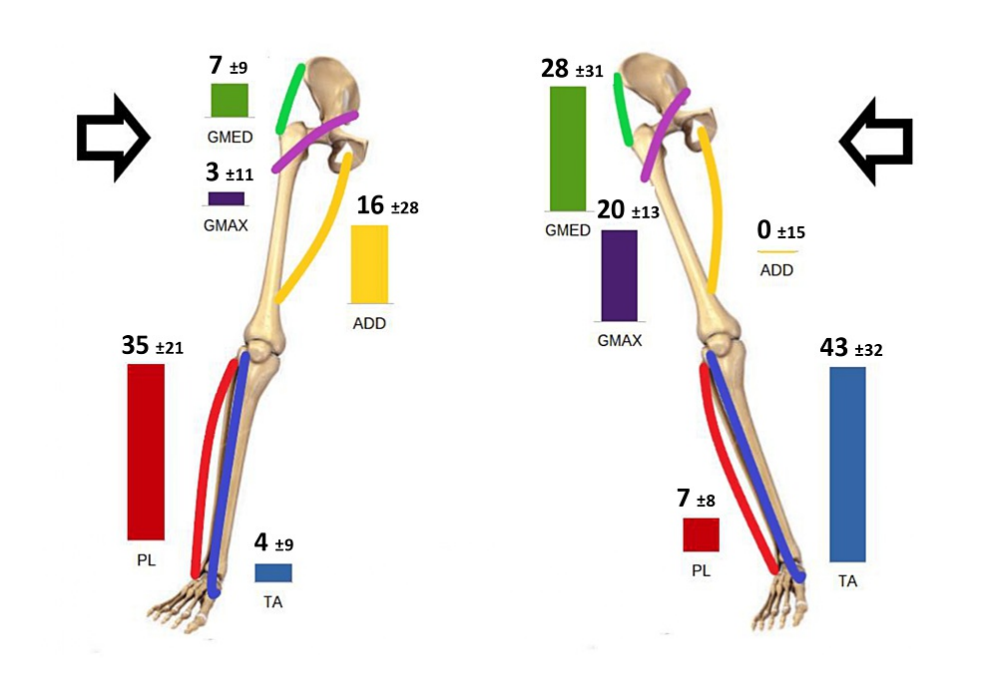
Average electrical peak response with standard deviation (expressed as a percentage of maximal contraction) to medial and lateral imbalance.

The average electrical peak responses are indicated in Figure 3.

The Pearson correlation coefficients of the analyzed muscles are presented in Table 2. The correlation was calculated between the curves shown in Figure 2. The sampling frequency was 1ms; therefore, 1500 values were considered for each curve. The p-value associated with each coefficient was always less than 0.001.

**Table 2:** Correlation coefficients. Correlation coefficients between each muscle of the ankle (tibialis anterior – TA, and peroneus longus – PL) and each of the hip (gluteus medius – GMED, gluteus maximus – GMAX, adductor longus – ADD), for the response to a medial and lateral imbalance in each subject (S1-S5).

MEDIAL IMBALANCE						
	S1	S2	S3	S4	S5	Average
TA-GMED	0.37	0.56	-0.24	0.79	0.70	0.44
TA-GMAX	0.11	0.52	0.08	0.68	-0.71	0.14
TA-ADD	0.64	0.60	0.79	0.72	-0.58	0.43
PL-GMED	0.35	0.33	0.61	0.91	0.80	0.60
PL-GMAX	-0.18	0.46	-0.42	0.93	0.79	0.32
PL-ADD	0.86	0.67	0.92	0.81	0.91	0.83
LATERAL IMBALANCE						
	S1	S2	S3	S4	S5	average
TA-GMED	0.92	0.89	0.93	0.88	0.84	0.89
TA-GMAX	0.82	0.93	0.89	0.77	0.90	0.86
TA-ADD	0.86	0.93	0.97	0.90	0.68	0.87
PL-GMED	-0.78	0.15	0.54	0.13	0.50	0.11
PL-GMAX	-0.65	0.12	0.53	0.38	0.37	0.15
PL-ADD	-0.82	-0.17	0.44	0.15	0.43	0.01

It was possible to identify some trends, especially regarding activation peaks. As an aid in comparing activation peaks at the level of the ankle (TA vs PL) and of the hip (ADD vs GMED, ADD vs GMAX), the p-values calculated with the t-test will be indicated in the following paragraphs as additional information. Anyway, given the small sample, the indication of the p-value is not intended as a conclusive statement of clinical or statistical significance.

Push from the right medial imbalance

At the ankle level, activation of PL prevailed over that of TA (p=0.01). S1 and S3 behaviors differed from the others: S3 significantly activated TA, but less than PL and only subsequently, and S1 had an important activation of PL (20-25%) even before the perturbation (he already used that muscle to maintain posture and increased its use to counteract the push).

At the hip level, there was generally an early ADD response, but with highly variable intensity. ADD peak tended to be higher than the GMED peak (p=0.45) and than the GMAX peak (p=0.30). The extreme cases were S2, which did not activate ADD at all, and S3, which had a sub-maximal contraction. GMAX and GMED were used more than ADD at rest, but they either responded with higher latency, or they responded with a lower intensity than ADD (S3).

The early response to a medial imbalance appears to be driven by PL and ADD, which react with lower latency and in a coordinated way. This also emerges from the observation of the correlation coefficients between ankle and hip muscles: the highest coefficient on average, 0.83, (and the highest in four out of five subjects) is that of PL-ADD.

Push from the left lateral imbalance

Considerations are similar and mirror those of the push from the right. At the ankle, TA prevailed over PL (p=0.04). However, a co-contraction was always present. A particular case was again that of S1, which, having PL already active, partially deactivated it after the stimulus.

At the hip, there was generally a modest ADD response (again S2 did not activate it at all) and a more significant one of the glutei. Three out of five subjects activated GMAX more than GMED (GMED peak > ADD peak with p=0.07; GMAX peak > ADD peak with p=0.03).

The response, therefore, seemed to be guided by TA and glutei, which had an early activation. The mean correlation of TA was high with respect to all hip muscles: ADD (0.87), GMED (0.89), and GMAX (0.86), but the functional contribution of ADD could be considered negligible, as its activation was very low.

## Discussion

This pilot study identified the activation of hip and ankle muscles as a response to medial and lateral imbalance during semi-unipodal stance. The intent was to verify if there was an activation of the spiral myofascial chains as hypothesized by Colonna [17].

Participation of the ankle muscles was highly repeatable and consistent. These results suggest, as it is for bipodalic stance [2], that an ankle strategy controls the balance in the first place. PL and TA activations were in agreement with the results obtained by Morey-Klapsing et al. [16]. The behavior of the hip muscles was more variable. However, a trend was identifiable: glutei tended to react more to a lateral imbalance and ADD to a medial one. Looking at hip and ankle activations globally, a prevalence of cross (or spiral)-activation could be detected (Figure 3).

This activation was consistent with the hypothesis formulated by Colonna [17]: TA and glutei (belonging to the posterior spiral chain) were prevalent in managing lateral imbalance, and PL and ADD, of the anterior spiral chain, were more relevant to compensate for the medial imbalance. This result may not be intuitive. For example, one might expect a completely lateral activation, both at the ankle and at the hip level (PL with the glutei), to counteract a medial imbalance.

If balance was achieved through spiral activations, it is reasonable to think that, for such an important and frequently used task, a spiral structure should be also present in the musculo-connective system. It is also reasonable that this structure includes the muscles used for that task. The anatomical findings about these spiral fascial connections [21] could be associated with this use. The connection through myofascial chains can mechanically constitute a guide to the movement that generates intrinsic coordination of the same, making it more efficient [23].

It is worth noting that, to study motor coordination more deeply, the movements of the whole body should have been considered. In fact, not only the supporting limb, but all body segments can be functional to maintain balance: head, trunk, pelvis, upper limbs, lower limb in suspension (or, as in this case, with less load). The displacement of each of these affects the displacement of the center of mass and produces forces of inertia. The activation of the glutei and ADD could be seen in this perspective: in the medial imbalance, the adductors allowed to load more of the left limb (which, at rest, was almost unloaded) and can be considered functional to a parachute reaction; in the lateral imbalance, however, the left limb was not available as a parachute. On the contrary, the latter tended to be lifted by the thrust, and the activation of the glutei tended to tilt the pelvis, lifting it further; from this position, the raised limb could be abducted, and the displacement of its mass to the left could help in keeping the center of pressure within the support base. These hypotheses were not analyzed in detail in this study and require kinematic and dynamic analysis to be verified.

If the balance were actually managed in the lower limb by means of spiral chains, this could have important clinical implications. For the recovery and prevention of an ankle inversion distortion trauma, it could be useful, for example, to associate the work on inversors with work on adductors, to strengthen them and make them more reactive. Indications could be drawn for the evaluation and treatment of the unstable equilibrium of the elderly and of many patients with neurological diseases. For example, a problem could be the lack of synergy in a chain, the lack of activation of it, or the weakness of it. Interesting questions may arise: is it possible to correlate the functionality of the spiral chains to the ability to maintain balance (can they be predictive of falls)? Is it possible to improve stability by acting on the peripheral systems (even passive ones) when the control ones are compromised? Is it possible to identify strategies to reinforce/stimulate/rebalance the spiral chains to increase their functionality? If the answer to these questions were to be affirmative, innovative therapeutic possibilities would open up.

The following limits were recognized. The destabilizing perturbation was performed manually, thus operator-dependent and potentially variable. Deep muscles (i.e., iliopsoas, piriformis, tibialis posterior) were excluded from the use of surface electromyography. The analysis was limited to the lower limb only, without joint dynamics and kinematics, which are, however, relevant for a more in-depth understanding of the motor strategy, its causes, and its effects. The number of participants was not adequate to provide statistically relevant results. No muscles were examined that did not have obvious action in the frontal plane: these muscles could have served as controls to verify that there was an actual difference in electrical response. The destabilization carried out was of a particular type, which could affect the type of response, and which could be different from other types of perturbation (e.g., on the foot or on the shoulder). Only healthy participants were included, so the results cannot be extended to pathological subjects, such as patients with neuromuscular disorders. If, as Horak et al. stated, the repertoire of motor programs is limited, then the strategies are likely to remain essentially the same [2]. However, the variability of responses seems to have indicated the opposite.

Future developments will be able to overcome these limitations by increasing the sample size, investigating additional muscles, and considering whole-body dynamics. It will also be appropriate to evaluate different methods of destabilization and the effect that these have on subjects with neuromotor pathologies.

## Conclusions

The method utilized in this study seemed to be appropriate to isolate the EMG response to imbalance and distinguish the different muscle responses to medial and lateral imbalance. These preliminary results were consistent with the literature at the ankle level and suggest the presence of a trend of synergies in the motor strategy. The muscle activations detected supported the hypothesis that the spiral myofascial chains of the lower limb are used to achieve semi-unipodal balance.
